# Sex differences in the characteristics and short-term prognosis of patients presenting with acute symptomatic pulmonary embolism

**DOI:** 10.1371/journal.pone.0187648

**Published:** 2017-11-06

**Authors:** Deisy Barrios, Raquel Morillo, Ina Guerassimova, Esther Barbero, Héctor Escobar-Morreale, Alexander T. Cohen, Cecilia Becattini, Victor Tapson, Roger Yusen, David Jimenez

**Affiliations:** 1 Respiratory Department, Hospital Ramón y Cajal and Medicine Department, Universidad de Alcalá (IRYCIS), Madrid, Spain; 2 Endocrinology Department, Hospital Ramón y Cajal and Medicine Department, Universidad de Alcalá (IRYCIS), Madrid, Spain; 3 Department of Haematological Medicine, Guys and St Thomas' NHS Foundation Trust, London, United Kingdom; 4 Internal and Cardiovascular Medicine, University of Perugia, Perugia, Italy; 5 Cedars-Sinai Medical Center, Los Angeles, California, United States of America; 6 Divisions of Pulmonary and Critical Care Medicine and General Medical Sciences, Washington University School of Medicine, St. Louis, Missouri, United States of America; Medical University Innsbruck, AUSTRIA

## Abstract

**Background:**

We sought to examine sex-related differences in the characteristics and outcome in patients presenting with acute symptomatic pulmonary embolism (**PE**).

**Methods:**

We conducted a retrospective cohort study of 2,096 patients diagnosed with acute PE. The characteristics were recorded at presentation. Treatment was at the discretion of patients’ physicians. The primary study outcome, all-cause mortality, and the secondary outcomes of PE-specific mortality, recurrent venous thromboembolism, and major bleeding were assessed during the first month of follow-up after PE diagnosis.

**Results:**

Overall, the women were older than the men and had significantly higher rates of immobilization. They had significantly lower rates of chronic obstructive pulmonary disease and cancer. Women had a higher prevalence of syncope and elevated brain natriuretic peptide levels. Thirty-day all-cause mortality was similar between women and men (7.1% versus 6.2%; *P* = 0.38). Male gender was not independently significantly associated with PE-related death (adjusted odds ratio [**OR**] 1.02; 95% CI, 0.50 to 2.07; *P* = 0.96). Restricting the analyses to haemodynamically stable patients (n = 2,021), female gender was an independent predictor of all-cause (adjusted OR 1.56; 95% CI, 1.07 to 2.28; *P* = 0.02) and PE-specific mortality (adjusted OR 1.85; 95% CI, 1.02 to 3.33; *P* = 0.04). Compared with men, women were 2.05 times more likely to experience a major bleed.

**Conclusions:**

Women and men with PE had different clinical characteristics, presentation, and outcomes. Women receiving anticoagulation have a significantly higher risk of major bleeding, suggesting the need for careful monitoring of anticoagulant intensity in women.

## Introduction

Acute pulmonary embolism (**PE**) is a potentially life-threatening disease, spanning a wide spectrum of clinical outcomes [1]. Classification of risk drives treatment decisions for patients with acute symptomatic PE. Haemodynamically stable patients with preserved right ventricular (**RV**) size and function are classified as low-risk patients and have an excellent short-term prognosis once therapeutic levels of anticoagulation therapy are established [2, 3]. In contrast, haemodynamically unstable patients are at high risk of death from worsening RV failure and cardiogenic shock, with a hospital mortality rate >15% [4]. Approximately one quarter of haemodynamically stable patients with PE show imaging or biomarker evidence of RV dilatation or dysfunction, with mortality rates ranging from 3% to 15% [5].

Sex differences in arterial disease have received considerable attention [6–8], but few studies have dealt with sex differences in venous thromboembolism (**VTE**) [9]. Furthermore, studies of patients with proven acute PE have shown conflicting data regarding the association between sex and adverse outcomes rates [10–13]. In a large study of 276,484 discharges with acute PE identified form the Nationwide Inpatient Sample (**NIS**), there was significantly higher in-hospital mortality in women compared to men [10]. In contrast, Aujesky and colleagues found that male patients had a higher risk of 30-day death compared to female patients [11]. Two smaller studies did not find a significant association between sex and prognosis [12, 13].

Sex differences in presentation and clinical course may dictate different approaches to detection and management. Given the limited information available, we used data from a prospective observational registry to assess sex-based differences in presentation and outcome of patients with objectively confirmed acute symptomatic PE.

## Methods

The Ethics Review Board at the Ramón y Cajal Hospital, Madrid, Spain, approved the study. The study methods and results are reported in accordance with the STROBE guidelines [14].

### Data source

The Ramón y Cajal Pulmonary Embolism Registry, initiated in 2003, has been described previously [15]. This registry includes information on the risk factors, baseline characteristics, medications, and complications for adult patients with acute symptomatic PE. Each patient provides informed consent for inclusion in the registry, and all patients who have confirmed PE are enrolled. Patients with PE between January 1, 2003, and December 31, 2016, were included in this study.

We confirmed the diagnosis of PE by objective testing that consisted of an intraluminal filling defect in segmental or larger vessels on computerized tomography pulmonary angiography (**CTPA**) [16], a high probability ventilation-perfusion (**V/Q**) scintigraphy [17], or a lower limb venous compression ultrasonography positive for proximal DVT in a patient with chest symptoms [18].

### Outcomes

This study used all-cause mortality through 30 days after initiation of treatment as the primary endpoint, and 30-day PE-related mortality, recurrent VTE, and major bleeding as secondary endpoints.

Two investigators (authors D.B. and D.J.) independently adjudicated the cause of deaths as (1) fatal PE, or (2) death from other causes. For deaths confirmed by autopsy, or those following a clinically severe PE, in the absence of any alternative diagnosis, the adjudicators judged death as due to fatal PE. Disparities in cause of death were resolved by consensus.

Investigators defined recurrent DVT as the appearance of a new noncompressible vein segment, or a 4-mm or more increase in the diameter of a thrombus on complete compression ultrasound (**CCUS**) [19]; recurrent PE as the presence of a new perfusion defect involving 75% or more of a lung segment on V/Q scintigraphy, or a new intraluminal filling defect or an extension of a previous filling defect on CTPA [16]; and major bleeding episodes as those that required a transfusion of at least 2 units of blood, were retroperitoneal, spinal or intracranial, or were fatal [20]. Two investigators (authors D.B. and D.J.) adjudicated all suspected events.

### Treatment and follow-up

The study did not require strict adherence to a standardized treatment protocol. Prior to hospital discharge, patients were instructed to contact the investigators if symptoms of recurrent PE or new or recurrent DVT occurred. Patients with suspected VTE were instructed to undergo diagnostic testing without delay. Otherwise, patients were seen in the investigators’ outpatient clinic at the end of the 1-month follow-up period.

### Statistical analyses

Discrete variables were presented as frequencies and percentages, and group comparisons were performed using the chi-square or Fisher’s exact tests. Continuous variables were presented as mean values with standard deviations (**SD**), and group comparisons were performed with the Mann-Whitney U test. To estimate the outcomes of time to death and time to VTE recurrence and bleeding, Kaplan-Meier probabilities were computed [21], and differences between the groups were assessed with the log-rank test. The impact of the variable of gender (i.e., female vs. male) on 30-day all-cause mortality was evaluated using univariable and multivariable logistic regression. For construction of the full models, we considered variables with imbalance between the groups at baseline for inclusion. During model construction, we did not remove variables that showed evidence of confounding (i.e., the coefficient of the variable changed by more than 10% when removed from the full model) for the effect of gender on the outcome undergoing analysis. To test the robustness of the models, the effects of excluding haemodynamically unstable patients were assessed. Statistical significance was defined as a two-tailed *P*-value of <0.05 for all analyses. Analyses were performed using SPSS, version 18.0 for the PC (SPSS, Inc. Chicago, IL, USA).

## Results

### Study sample

**Fig 1:**
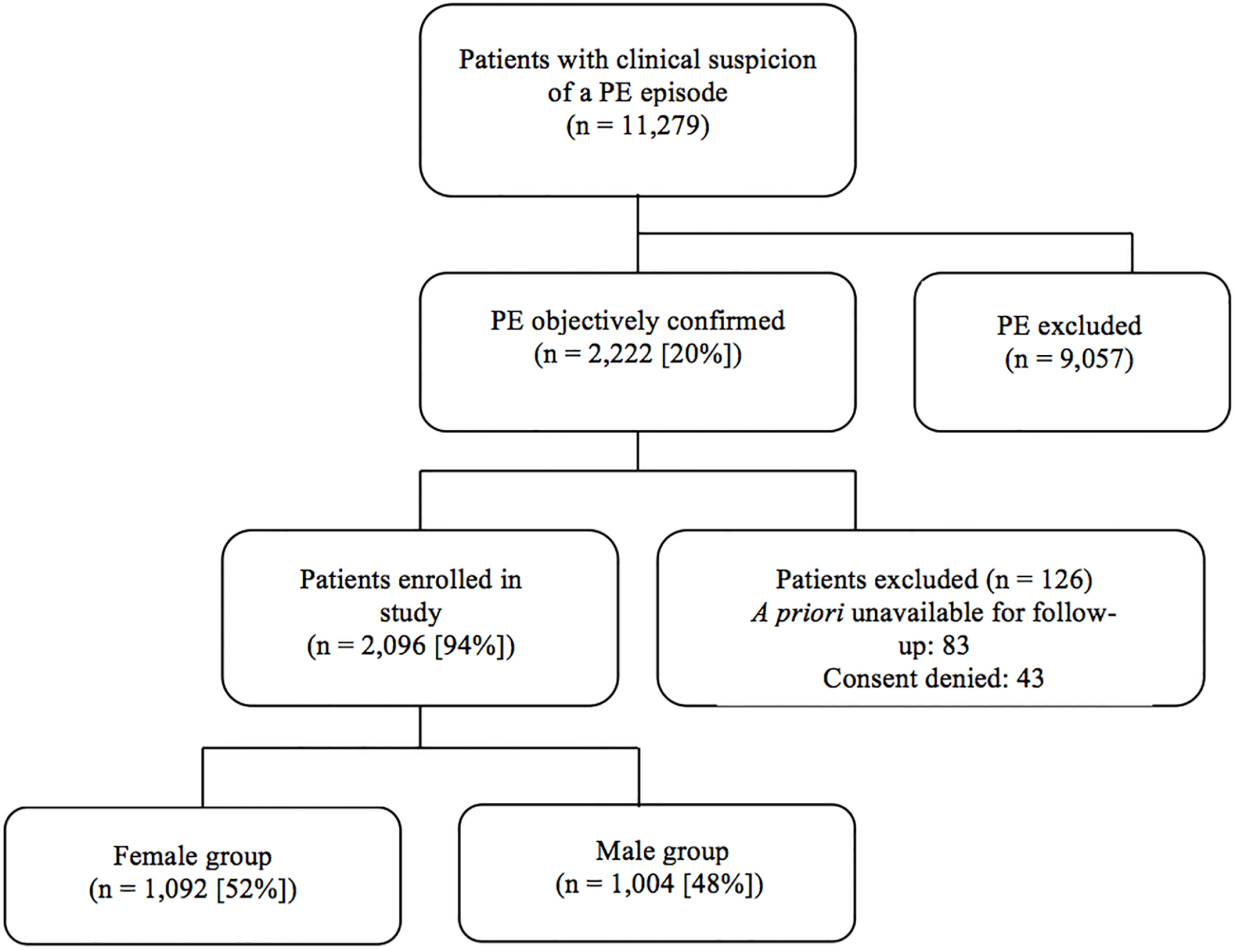
Patient flow diagram.

Of the 11,279 patients with a clinical suspicion of a PE screened for the study, 19.7% (2,222 of 11,279 patients) had an objective diagnosis of PE. Of these, 5.7% (126 of 2,222 patients) were excluded because they were unavailable for follow-up (n = 83), or refused to give informed consent (n = 43) (Fig 1). The eligible study cohort of 2,096 patients included 1,092 women and 1,004 men. In the vast majority of patients, PE was diagnosed by a high-probability V/Q scan (33%; 688 of 2,096 patients) and/or a positive PE-protocol CT (70%; 1,476 of 2,096 patients). Diagnosis was based on CCUS results in 55 of the 2,096 patients (2.6%; 95% CI, 2.0% to 3.4%). Some patients received multiple diagnostic tests.

### Baseline characteristics

Women with acute PE differed significantly from men in preexisting medical conditions, and in relevant clinical, physiologic and laboratory parameters. As shown in Table 1, women were older and had a higher prevalence of immobilization compared to men. Men had a higher prevalence of chronic obstructive pulmonary disease (**COPD**), cancer, and renal failure compared to women. Women had a higher prevalence of syncope and elevated brain natriuretic peptide (**BNP**) levels (Table 1). With respect to similarities, a history of VTE and recent surgery were almost identical in women and men, as was the frequency of dyspnea, chest pain, tachycardia, hypoxemia and hypotension. The two groups had similar proportions of patients treated with inferior vena cava filters or thrombolytic therapy (Table 1).

**Table 1:** Baseline characteristics and treatment information for patients with acute symptomatic pulmonary embolism.

	*All patients N = 2*,*096*	*Male group N = 1*,*004*	*Female group N = 1*,*092*	*P value*
**Clinical characteristics,**				
Age, years (mean ± SD)	68.7 ± 16.6	66.6 ± 16.2	70.6 ± 16.8	< 0.001
Age > 65 years	1394 (67%)	602 (60%)	792 (73%)	< 0.001
Body mass index, Kg m^-2^ (mean ± SD)	27.2 ± 6.4	26.8 ± 4.4	27.5 ± 7.6	0.05
Delays in diagnosis, days (mean ± SD)	5.0 ± 11.2	4.9 ± 8.2	5.0 ± 13.4	0.81
**Risk factors for VTE,**				
History of VTE	249 (12%)	121 (12%)	128 (12%)	0.84
Cancer^†^	432 (21%)	232 (23%)	200 (18%)	<0.01
Recent surgery^‡^	188 (9.0%)	83 (8.3%)	105 (9.6%)	0.28
Immobilization^y^	412 (20%)	160 (16%)	252 (23%)	< 0.001
**Comorbid diseases,**				
Recent major bleeding^‡^	70 (3.3%)	41 (4.1%)	29 (2.7%)	0.09
Chronic obstructive pulmonary disease (COPD)	169 (8.1%)	136 (14%)	33 (3.0%)	< 0.001
Congestive heart failure	116 (5.5%)	46 (4.6%)	70 (6.4%)	0.07
**Clinical symptoms and signs at presentation**				
Syncope	300 (14%)	125 (12%)	175 (16%)	0.02
Chest pain	936 (45%)	454 (45%)	482 (44%)	0.66
Dyspnea	1,514 (72%)	717 (71%)	797 (73%)	0.44
Heart rate ≥ 110/minute	426 (20%)	188 (19%)	238 (22%)	0.08
Arterial oxyhemoglobin saturation < 90%	563 (27%)	259 (26%)	304 (28%)	0.30
SBP < 90 mm Hg	75 (3.6%)	36 (3.6%)	39 (3.6%)	0.99
Concomitant DVT (n = 1,630)	915/1,630 (6%)	461/784 (59%)	451/846 (53%)	0.02
**Cardiac biomarkers, n (%)**				
BNP > 100 pg/mL (n = 805)	379 (47%)	163 (40%)	216 (54%)	< 0.001
cTnI > 0 ng/mL (n = 1,564)	460 (29%)	202 (27%)	258 (31%)	0.06
**sPESI**				
Low-risk	638 (30%)	318 (32%)	320 (29%)	0.25
High-risk	1,458 (70%)	686 (68%)	772 (71%)	0.25
**Laboratory findings**				
Creatinine levels > 1.5 mg/dL	91 (11%)	106 (11%)	133 (12%)	0.01
**Treatment**				
Thrombolytic therapy	91 (4.3%)	54 (5.4%)	37 (3.4%)	0.19
Insertion of an IVC filter	49 (2.3%)	18 (1.8%)	31 (2.8%)	0.15

**Abbreviations**: SD, standard deviation; VTE, venous thromboembolism; COPD, chronic obstructive pulmonary disease; SBP, systolic blood pressure; BNP, brain natriuretic peptide; cTnI, cardiac troponin I; sPESI, simplified Pulmonary Embolism Severity Index; IVC, inferior vena cava.

^†^Active or under treatment in the last year

^‡^In the previous month

^y^Immobilized patients are defined in this analysis as non-surgical patients who had been immobilized (i.e., total bed rest with bathroom privileges) for ≥4 days in the month prior to PE diagnosis

### Outcomes

#### Mortality

**Fig 2:**
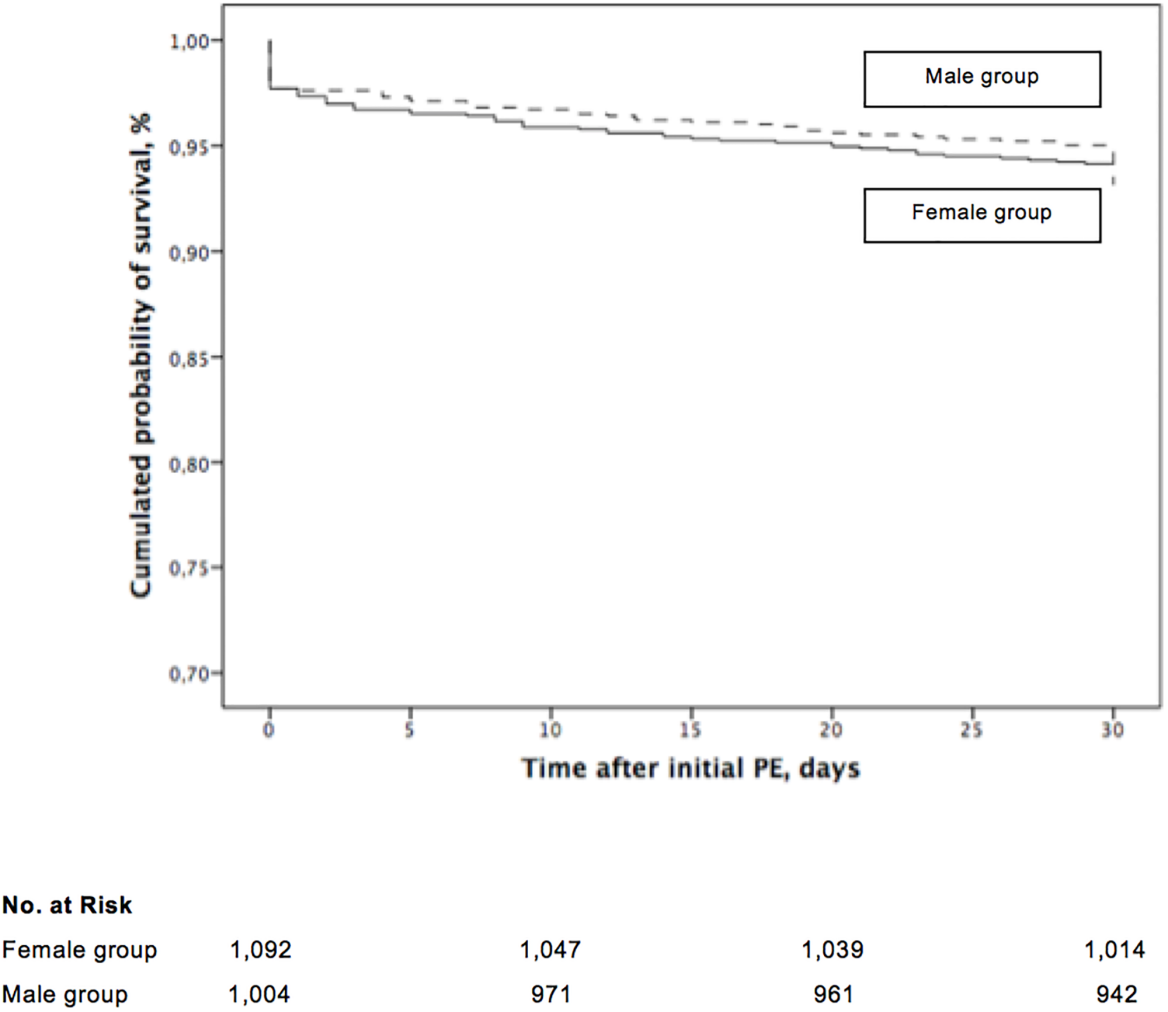
Mortality of patients with acute symptomatic pulmonary embolism, stratified by gender from the time of diagnosis.

Mortality data were available for all patients at the conclusion of the study. Overall, 140 out of 2,096 patients died (6.7%; 95% confidence interval [**CI**], 5.6% to 7.8%) during the first month of follow-up. Sixty-two patients (62 of 2,096 patients; 3.0%; 95% CI, 2.3% to 3.8%) died from definite (n = 23) or possible PE (n = 39), whereas other deaths were caused by cancer (1.5%; 31 of 2,096 patients), infection (0.9%; 19 of 2,096 patients), major bleeding (0.3%; 7 of 2,096 patients), other diseases (0.8%; 17 of 2,096 patients), and unknown causes (0.2%; 4 of 2,096 patients). Seventy-eight deaths (78 of 1,092 patients; 7.1%; 95% CI, 5.7% to 8.8%) occurred in the group of female patients entering the study with acute PE, whereas 62 deaths (62 of 1,004 patients; 6.2%; 95% CI, 4.8% to 7.8%) occurred in the group of male patients (absolute difference 0.9%; 95% CI of the absolute difference, -1.2% to 3.1%; *P* = 0.38). Female patients with acute PE had a similar cumulative mortality compared to male patients with acute PE (*P* = 0.37, log rank test; Fig 2).

In multivariate analyses, patients with congestive heart failure (odds ratio [**OR**] 3.52; 95% CI, 1.69 to 7.34; *P* < 0.01), recent major bleeding (OR 4.10; 95% CI, 1.77 to 9.51; *P* < 0.01), presence of concomitant DVT (OR 1.69; 95% CI, 1.04 to 2.77; *P* = 0.04), systolic blood pressure < 90 mmHg (OR 3.30; 95% CI, 1.27 to 8.60; *P* < 0.01), syncope (OR 0.35; 95% CI, 0.15 to 0.86; *P* = 0.02), and cancer (OR 5.35; 95% CI, 3.31 to 8.66; *P* < 0.001), but not male gender (OR 1.00; 95% CI, 0.63 to 1.57; *P* = 0.99) were significantly more likely to die during follow-up (Table 2). After adjustment, male gender was not independently significantly associated with PE-related death (adjusted OR 1.02; 95% CI, 0.50 to 2.07; *P* = 0.96) (Table 3). Restricting the analyses to haemodynamically stable patients (i.e., systolic blood pressure ≥ 90 mmHg) (n = 2,021), female gender was an independent predictor of all-cause (female gender, adjusted OR 1.56; 95% CI, 1.07 to 2.28; *P* = 0.02) and PE-specific mortality (female gender, adjusted OR 1.85; 95% CI, 1.02 to 3.33; *P* = 0.04) (Table 4).

**Table 2:** Unadjusted and adjusted odds ratios for overall mortality in patients with acute symptomatic pulmonary embolism.

*Risk factor*	*Unadjusted OR (95% CI)*	*P value*	*Adjusted OR (95% CI)*	*P value*
Age, per year	1.02 (1.01–1.04)	< 0.001	1.02 (1.00–1.03)	0.08
Male gender	0.86 (0.61–1.21)	0.38	1.00 (0.63–1.57)	0.99
COPD	1.64 (0.96–2.80)	0.07	-	-
Congestive heart failure	2.39 (1.37–4.18)	< 0.01	3.52 (1.69–7.34)	< 0.01
Recent major bleeding^†^	3.06 (1.60–5.84)	< 0.01	4.10 (1.77–9.51)	< 0.01
Presence of DVT	1.78 (1.19–2.69)	< 0.01	1.69 (1.04–2.77)	0.04
SBP < 90 mmHg	3.79 (2.09–6.87)	< 0.001	3.30 (1.27–8.60)	0.01
Heart rate ≥ 110 bpm	1.51 (1.02–2.22)	0.04	1.13 (0.65–1.98)	0.66
Arterial oxyhemoglobin saturation < 90%	1.97 (1.38–2.80)	< 0.001	1.64 (1.01–2.68)	0.05
Dyspnea	1.83 (1.17–2.85)	< 0.01	0.98 (0.55–1.75)	0.95
Chest pain	0.51 (0.35–0.74)	< 0.001	0.68 (0.41–1.12)	0.13
Syncope	0.49 (0.26–0.92)	0.03	0.35 (0.15–0.86)	0.02
Cancer^‡^	3.63 (2.56–5.16)	< 0.001	5.35 (3.31–8.66)	< 0.001
Immobilization^y^	1.51 (1.10–2.06)	0.01	1.63 (0.94–2.82)	0.08
Insertion of an IVC filter	1.61 (0.63–4.12)	0.32	-	-

**Abbreviations**: OR, odds ratio; CI, confidence interval; COPD, chronic obstructive pulmonary disease; DVT, deep vein thrombosis; SBP, systolic blood pressure; IVC, inferior vena cava.

^†^In the previous month.

^‡^Active or under treatment in the last year.

^y^Immobilized patients are defined in this analysis as non-surgical patients who had been immobilized (i.e., total bed rest with bathroom privileges) for ≥4 days in the month prior to PE diagnosis.

N = 2,096 evaluated, with 62 deaths. Final model chi square = 31.81, *P* < 0.001

**Table 3:** Unadjusted and adjusted odds ratios for PE-related death in patients with acute symptomatic pulmonary embolism.

*Risk factor*	*Unadjusted OR (95% CI)*	*P value*	*Adjusted OR (95% CI)*	*P value*
Age, per year	1.04 (1.02–1.06)	< 0.001	1.04 (1.01–1.07)	0.02
Male gender	0.73 (0.43–1.22)	0.23	1.02 (0.50–2.07)	0.96
COPD	1.23 (0.52–2.90)	0.64	-	-
Congestive heart failure	1.87 (0.79–4.44)	0.15	-	-
Recent major bleeding^†^	2.05 (0.72–5.81)	0.18	-	-
Presence of DVT	2.25 (1.24–4.09)	< 0.01	2.92 (1.25–6.83)	0.01
SBP < 90 mmHg	6.64 (3.30–13.34)	< 0.001	4.89 (1.71–14.02)	< 0.01
Heart rate ≥ 110 bpm	2.06 (1.20–3.52)	< 0.01	1.17 (0.51–2.70)	0.71
Arterial oxyhemoglobin saturation < 90%	2.82 (1.70–4.69)	< 0.001	1.71 (0.82–3.53)	0.15
Dyspnea	1.46 (0.79–2.72)	0.23	-	-
Chest pain	0.58 (0.34–1.00)	0.05	-	-
Syncope	0.88 (0.42–1.87)	0.75	-	-
Cancer^‡^	2.02 (1.18–3.46)	0.01	1.82 (0.81–4.09)	0.15
Immobilization^y^	1.50 (1.08–2.08)	0.02	1.95 (0.91–4.17)	0.08
Insertion of an IVC filter	1.41 (0.33–5.93)	0.64	-	-

**Abbreviations**: OR, odds ratio; CI, confidence interval; COPD, chronic obstructive pulmonary disease; DVT, deep vein thrombosis; SBP, systolic blood pressure; IVC, inferior vena cava.

^†^In the previous month.

^‡^Active or under treatment in the last year.

^y^Immobilized patients are defined in this analysis as non-surgical patients who had been immobilized (i.e., total bed rest with bathroom privileges) for ≥4 days in the month prior to PE diagnosis.

N = 2,096 evaluated, with 140 deaths. Final model chi square = 98.00, *P* < 0.001

**Table 4:** Adjusted odds ratios for all-cause and PE-specific mortality in haemodynamically stable patients with acute symptomatic pulmonary embolism.

*Risk factor*	*All-cause mortality Adjusted OR (95% CI)*	*P value*	*PE-specific mortality Adjusted OR (95% CI)*	*P value*
Male gender	0.64 (0.44–0.93)	0.02	0.54 (0.30–0.98)	0.04
Recent major bleeding^†^	3.07 (1.44–6.54)	< 0.01	2.96 (1.02–8.60)	0.05
Heart rate ≥ 110 bpm	1.49 (0.97–2.29)	0.07	1.91 (1.04–3.50)	0.04
Syncope	0.32 (0.14–0.74)	< 0.01	0.39 (0.12–1.27)	0.12
Cancer^‡^	4.08 (2.80–5.95)	< 0.001	2.09 (1.15–3.80)	0.02

**Abbreviations**: OR, odds ratio; CI, confidence interval; COPD, chronic obstructive pulmonary disease; DVT, deep vein thrombosis; SBP, systolic blood pressure; IVC, inferior vena cava.

^†^In the previous month.

^‡^Active or under treatment in the last year.

#### Recurrent venous thromboembolism

All surviving patients returned for follow-up. Eighty-nine (4.2%) of the 2,096 patients had clinically suspected recurrent VTE during follow up, and symptomatic VTE was objectively confirmed in 38 patients in the cohort (38 of 2,096 patients; 1.8%; 95% CI, 1.3% to 2.5%). Twenty-eight (1.3%; 95% CI, 0.9% to 1.9%) of 2,096 patients had recurrent symptomatic PE and 10 (0.5%; 95% CI, 0.2% to 0.9%) of 2,096 patients had symptomatic DVT (9 proximal and 1 distal).

**Fig 3:**
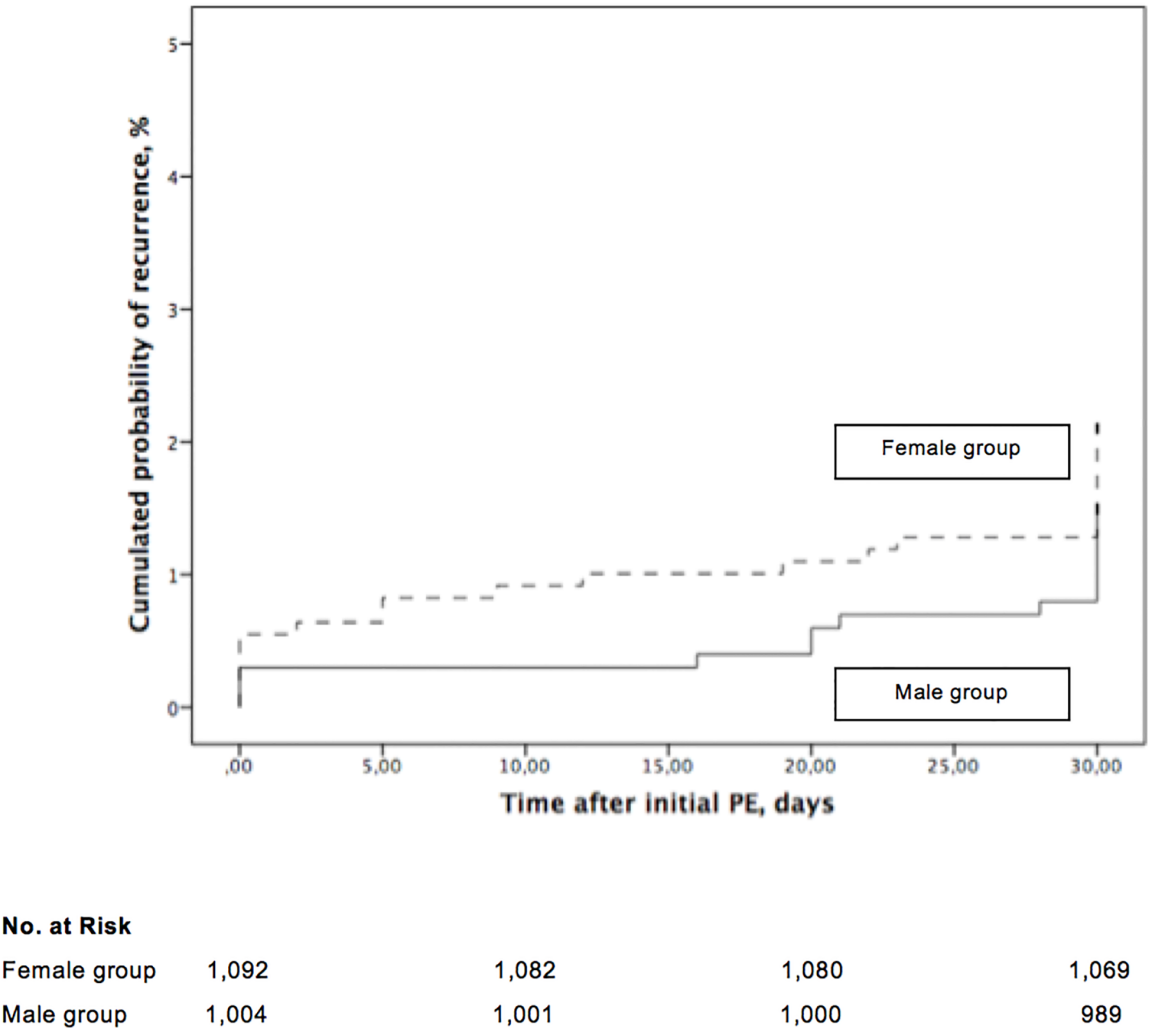
Recurrent symptomatic venous thromboembolism in patients with acute symptomatic pulmonary embolism, stratified by gender from the time of diagnosis.

Of the female patients, 23 (23 of 1,092 patients; 2.1%; 95% CI, 1.3% to 3.1%) had a symptomatic VTE recurrence during follow-up, whereas 15 (15 of 1,004 patients; 1.5%; 95% CI, 0.8% to 2.5%) of the male patients experienced a symptomatic VTE recurrence (absolute difference 0.6%; 95% CI of the absolute difference, -0.6% to 1.8%; *P* = 0.33). The cumulative incidence of symptomatic VTE recurrence was not different in men compared to women (*P* = 0.29, log rank test; Fig 3).

#### Major bleeding

**Fig 4:**
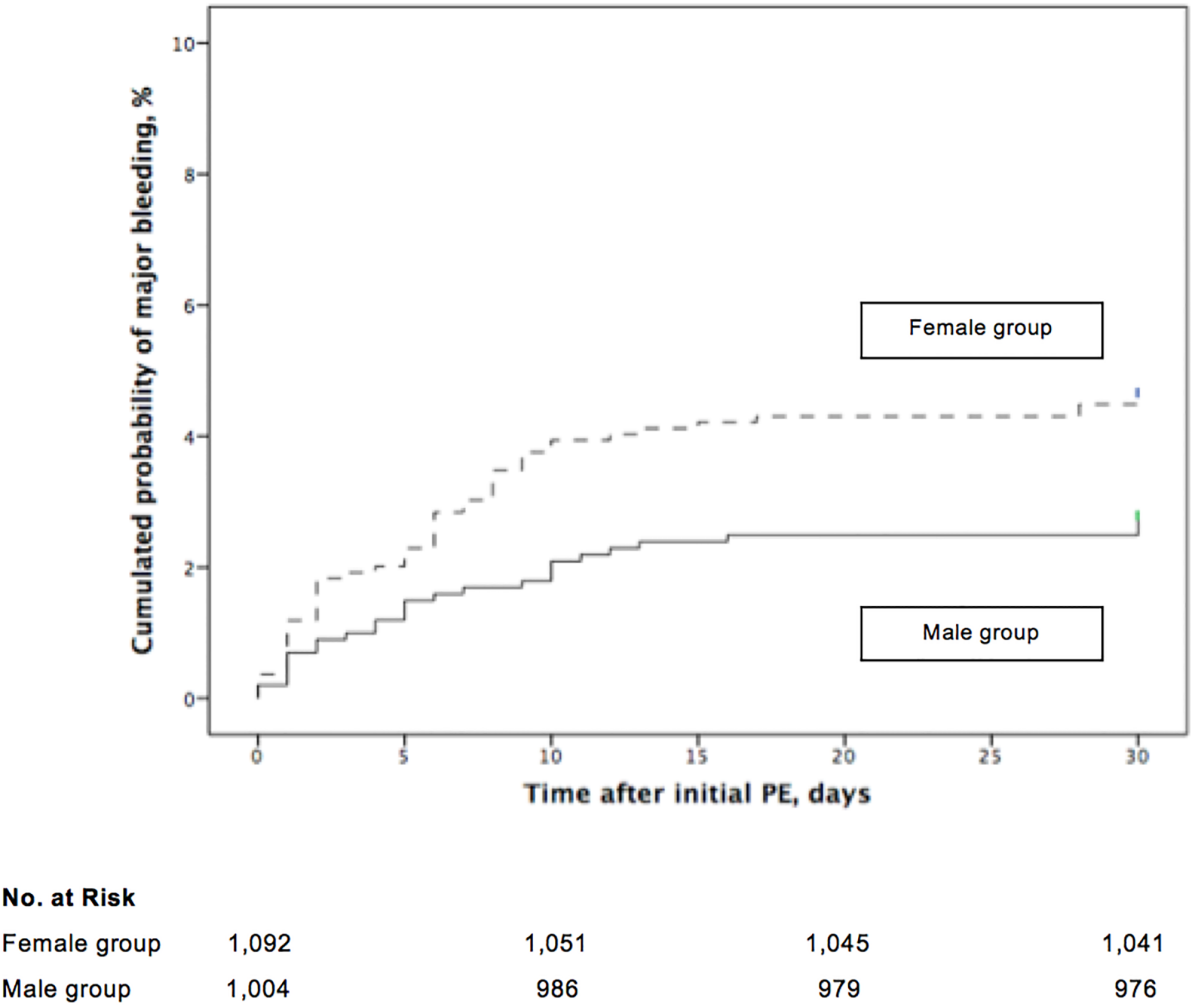
Major bleeding in patients with acute symptomatic pulmonary embolism, stratified by gender from the time of diagnosis.

Major bleeding was objectively confirmed in 79 patients in the cohort (79 of 2,096 patients; 3.8%; 95% CI, 3.0% to 4.7%). Of the female patients, 51 (51 of 1,092 patients; 4.7%; 95% CI, 3.5% to 6.1%) had a major bleeding during follow-up, whereas only 28 (28 of 1,004 patients; 2.8%; 95% CI, 1.9% to 4.0%) of the male patients experienced a major bleeding (absolute difference 1.9%; 95% CI of the absolute difference, 0.2% to 3.5%; *P* = 0.03). In the multivariate analysis, low body mass index was the only confounding variable for the association between female gender and major bleeding during follow-up (adjusted OR 2.05; 95% CI, 1.09 to 3.88; *P* = 0.03). The cumulative incidence of major bleeding was significantly higher in female patients compared to male patients (*P* = 0.02, log rank test; Fig 4). Separation between major bleeding curves occurred soon after the diagnosis of PE at study entry.

## Discussion

This study showed that women with acute symptomatic PE had a similar short-term risk for all-cause death, PE-related death, and recurrent VTE than men, after adjusting for potential confounders. However, among haemodynamically stable patients, the risk of death was about 1.6 times higher in women than in men. The risk of major bleeding among women was about 2.1 times higher than in men.

Consistent with our findings, the RIETE investigators reported that women who had PE were older than men and were more likely to have a history of congestive heart failure and immobilization [9]. Previous studies reported a significant sex difference in the distribution of thromboembolic events in patients with heart failure, with a greater frequency of PE in women [22, 23]. The women in our study were less likely to have COPD and cancer, but they were significantly more likely than the men to have myocardial stretch, as was also found in previous studies [9, 10]. This finding may relate to the higher percentage of women having a history of heart failure.

Haemodynamic status at the time of presentation with acute PE has the strongest prognostic implications for short-term mortality [1]. Though prior evidence suggests that women with PE are more likely to present with hypotension, hypoxemia, or right ventricular overload [9], the overall incidence of all-cause mortality, PE-related death, and recurrent VTE did not vary by sex in our study. Conflicting data exist regarding the association between sex and the risk of death in patients with acute symptomatic PE. A study of 10,354 patients that had acute PE showed an association between male sex and all-cause mortality [11]. In contrast, a larger study of 276,484 discharges with acute PE from the Nationwide Inpatient Sample found that women with PE had poorer outcomes (i.e., higher inhospital mortality) than men [10]. Intriguingly, we found that haemodynamically stable women were 1.6 times more likely to die during follow-up compared to men. Data from those studies in conjunction with our results may explain the variable prognostic findings of sex. Female sex appeared to have better predictive ability for mortality in studies that had lower rates of haemodynamically compromised patients. Our study’s large sample size, the adjustment for potential confounders, and the robustness of the findings provides evidence supporting the concept that sex is a predictor of all-cause death in PE patients without significant hypotension. The underlying mechanisms of the increased mortality risk associated with female gender in stable patients with acute PE remain to be elucidated. Difference between the sexes may relate to differences in thrombotic and fibrinolytic activity [24], or differences in the extent of disease in the lung, since our study found a lower frequency of concomitant DVT in women compared with men.

Our results suggest that women have a higher risk of major bleeding. Previous studies that enrolled patients with VTE who received vitamin K antagonists or direct oral anticoagulants have reported an increased rate of bleeding in women [9, 25]. A recent meta-analysis demonstrated marginally higher bleeding rates among women receiving anticoagulation for VTE compared to men [26]. Since the higher rate of major bleeding may not be attributed to differences in age, body mass index, renal failure, or treatment dosing between women and men, measures to prevent bleeding should be considered in female patients taking care not to compromise efficacy.

This study contains some methodological limitations. This single center study of patients that presented to a tertiary care urban emergency department may not generalize to other settings. Because this registry does not consistently collect data on the quality of anticoagulation (i.e., serial INR measurements) during follow-up, we cannot say whether the (lack of) association between gender and outcomes in our study may have been due to differences in anticoagulation quality. Finally, although this is a large study and the data in this registry allowed us to adjust for a number of key variables, the possibility of residual confounding still remains.

In conclusion, the results of this study of patients with acute symptomatic PE suggest that women, compared to men, have an increased risk of all-cause mortality (if not significantly hypotensive) and major bleeding over 1-month of follow-up. Further research is needed to determine which factors account for these significant differences in outcomes.
